# Revealing the dynamic changes in the metabolites and sensory quality of Citri Reticulatae Pericarpium during aging using feature-based molecular networks and metabolomics

**DOI:** 10.1016/j.fochx.2025.102884

**Published:** 2025-08-07

**Authors:** Kunli Xu, Sen Mei, Jiahuan Liu, Zirui Guo, Fanyu Meng, Yanbo Wang, Bei Wang

**Affiliations:** a Key Laboratory of Geriatric Nutrition and Health (Beijing Technology and Business University), Ministry of Education, Beijing 100048, China; b School of Food and Health, Beijing Technology and Business University, Beijing 100048, China; c State Key Laboratory of Food Science and Resources, Nanchang University, Nanchang 330047, China

**Keywords:** Citri Reticulatae Pericarpium, Non-volatile compounds, Sensory evaluation, Electronic tongue, Feature-based molecular network

## Abstract

The contribution of non-volatile metabolites to the unique flavor formation in Citri Reticulatae Pericarpium (CRP) during aging remains poorly characterized. This study employed sensory evaluation, electronic tongue (E-tongue) assessment, untargeted metabolomics in conjunction with feature-based molecular networks (FBMN) to elucidate flavor differentials and metabolic mechanisms during the aging process of CRP. The results showed that extended aging time enhanced the bitterness, astringency, aftertaste-bitterness, aftertaste-astringency, and sweetness of the CRP. FBMN tool used for the first time to annotate flavonoid components of CRP. Multivariate statistical analyses revealed that of the total 1092 identified metabolites, 189 differed significantly during aging. Combined with the results of KEGG pathway analysis, phenylpropanoid biosynthesis, flavonoid biosynthesis, and flavone and flavonol biosynthesis were the main enrichment pathways during the aging process of CRP aging. The results of this study clarified the mechanisms behind non-volatile metabolite fingerprint changes and flavor formation during the CRP aging process.

## Highlights

• The bitterness, astringency, and sweetness increased and the sourness decreased during CRP aging.
• The FBMN provided a framework for metabolite analysis, identification, and annotation.
• Statistical analysis identified 189 differential metabolites during CRP aging.
• Flavonoids played a significant role in flavor development during CRP aging.

## Graphical abstract

**Figure:**
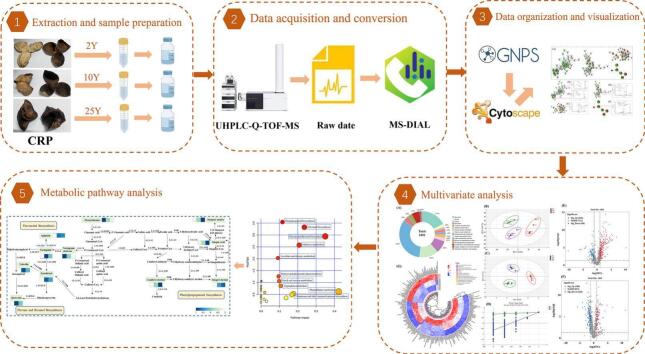

## 1 Introduction

Citri Reticulatae Pericarpium (CRP) is the dried, aged peel of mature *Citrus reticulata* Blanco and its cultivars, which has been used in China for thousands of years for its food and medicinal properties. CRP can be categorized into different aging periods according to distinct edible characteristics (Zhu et al., 2022). The aging duration of CRP significantly influences its quality. Notably, the variation in the sensory quality of CRP is primarily attributed to metabolite changes and accumulation during aging, and the sensory qualities and nutritional value of well-preserved CRP become more pronounced with extended aging, and the improvement in quality of CRP is primarily attributed to the accumulation of bioactive compounds (Shen et al., 2024). CRP offers excellent active antioxidant properties, which help stabilize blood glucose, cholesterol, and liver lipid levels in the body (Zou et al., 2022). Thus far, research on CRP during different aging processes focuses on determining and analyzing active ingredients, pharmacological effects (Fu et al., 2025), and volatile flavor compounds (Jiang, et al., 2023; Liu et al., 2024). However, minimal studies have used metabolomics techniques to explore the mechanism behind the flavor contribution of non-volatile compounds during CRP aging.

Natural CRP aging is a complex process where flavor evolution is affected by microbial population structures, metabolic enzyme activity, storage conditions, aging duration, and chemical composition changes (Huang, Hong, et al., 2024). During this process, the accumulated active ingredients are gradually transformed and released in a free form via physicochemical and microbial interactions, consequently substantially affecting their sensory quality. Previous studies have shown that volatile components significantly influence the sensory quality of CRPs aged for different periods. Our previous research on CRPs aged for 2, 5, 10, 15, 20, and 25 years revealed a gradual aroma change from floral and fruity attributes to herbal and woody characteristics as the aging duration was extended (Jiang et al., 2023). Based on this study, we will further investigate the effect of non-volatile components on the flavor of CRP. Flavonoids and phenols represent the main active components in CRP, while the levels of flavor components, such as amino acids, is relatively low, yielding an overall bland, bitter flavor (Shen et al., 2024). Flavonoid glycosides present significant bitter flavors with low detection thresholds. However, this bitterness is mitigated by interactions with other compatible flavor-enhancing active compounds (Li et al., 2023). Glycosylation and methylation during CRP aging considerably reduce the binding between the flavonoid glycoside and bitter receptors, improving their stability, reducing bitter intensity, and enhancing the flavor profile complexity (Huang, Liu, et al., 2024).

Research on the correlation between food flavor and changes in metabolite profiles can potentially inform food flavor improvement. Non-targeted metabolomics analysis using ultra-high performance liquid chromatography-quadrupole tandem time-of-flight mass spectrometry (UHPLC-Q-TOF-MS) is helpful for the large-scale evaluation of small-molecule intermediates and metabolites in food samples (García-Pérez et al., 2024). Identifying metabolites using this method relies on standard spectral libraries. However, most metabolites lack commercial reference standards, posing a challenge for systematic analysis, annotation, and identification (Shi et al., 2024). Data-driven approaches for chemical structure mining, such as Global Natural Products Social Molecular Networking (GNPS), have been established to address the limitations presented by databases (Nothias et al., 2020). GNPS is an open-access knowledge repository for community organizations to share, process, and characterize raw tandem mass spectrometry data. On the GNPS platform, FBMN considers a broader range of molecular information for metabolite profile categorization, including isotope integration patterns, secondary mass spectrometry data, and retention times. This enables the visualization and analysis of metabolites based on mass spectrometry fragmentation data and structural similarity assessment. FBMN offers significant benefits for rapid compound identification in complex mixtures and novel compound discovery (Li, Ma, et al., 2024). LC-MS/MS-based molecular network strategies are currently utilized in various fields, such as food functionality, safety, and quality assessment (Li, Wang, et al., 2023).

The formation of food flavor is a comprehensive result of the interaction between metabolite profiles and the human sensory system. Therefore, clarifying the characteristics of food metabolite profiles during dynamic changes is crucial for the in-depth analysis of the composition of food flavor components and their formation mechanisms. This study aims to achieve the following objectives: (i) synthesize and analyze the sensory quality differences during CRP aging using an electronic tongue (E-tongue) and sensory evaluation; (ii) characterize the CRP metabolite fingerprints at different aging stages using UHPLC-Q-TOF-MS based non-targeted metabolomics and FBMN to enhance metabolite annotation coverage; (iii) elucidate the CRP metabolite fingerprint variation at different aging stages via multivariate statistical analysis; and (iv) determine the metabolic pathways of differential compounds during CRP aging via the KEGG metabolic pathway. This study clarifies the mechanism of non-volatile compounds in flavor profiles, offering a novel scientific perspective and theoretical support for analyzing the complex flavor profile of CRP.

## 2 Materials and methods

### 2.1 Materials and reagents

The chromatography grade solvents, including acetonitrile (ACN), methanol (MeOH), and ammonium acetate (NH_4_AC), were obtained from Sigma Aldrich (St. Louis, MO, USA), while the formic acid (FA) was supplied by the Kermel Chemical Reagent Co. Ltd. (Tianjin, China). Deionized water (Milli-Q-Water) was prepared using a Millipore water purification system (Millipore, Burlington, MA).

The samples were consistent with our previous study (Jiang, et al., 2023). Samples of the same cultivar of peel (*Citrus reticulata* “Chachi”) harvested at the same location in the same season were sourced from the Ganguli Co. Ltd. (Xinhui, Guangdong, China). The peels were treated with the same method to ensure similar initial chemical compositions before aging. Previous study categorized CRPs aged for 2, 5, 10, 15, 20 and 25 years into young (2Y), middle (5Y and 10Y), and old (15Y, 20Y, and 25Y) aging stages based on the changes in aroma composition. In order to explore the non-volatile flavor components of this group of samples, *E*-tongue (SA402B, Insert Company, Japan) was used for the initial exploration of their taste, and the PCA results of the E-tongue were consistent with the previous studies in the classification of CRP (Fig. S1). Therefore, this study selected a group of samples (2Y, 10Y, and 25Y) from the young, middle, and old aging stages for non-volatile flavor component analysis.

### 2.2 Sensory evaluation of the CRP at different aging stages

For the subsequent experiments, 3.0 g of CRP was weighed into 200 mL of boiling water, immersed for 3 min, removed, and immersed again in 200 mL of boiling water for 3 min. The solution was filtered and evaluated using an E-tongue according to a previous assay by our research group (Xu et al., 2024). The E-tongue was equipped with six sensor probes for taste measurements: sourness (CAO), astringency (AE1), bitterness (C00), saltiness (CT0), umami (AAE), and sweetness (GL1). The aftertaste-astringency, aftertaste-bitterness, and richness of the CRP were also determined. The sensors were cleaned using a three-step method. First, the sensors were cleaned in a cationic or anionic solution for 90 s, followed by a contrasting solution for 120 s. The samples were tested for 30 s to determine the taste values of the CRP at different aging stages.

Twelve experienced sensory panelists (four males and eight females between 22 and 42 years old) from the College of Food and Health, Beijing Technology and Business University (BTBU) performed the quantitative descriptive analysis (QDA) of the CRP according to the GB/T 39625-2020 standard. Reference standards corresponding to each sensory attribute was shown in Table S1. The sensory attributes of the different aged CRP samples were evaluated using a 6-point scale (0, 3, 5 indicated zero, medium, and high intensity, respectively. Resolution was 0.5). (Beijing Technology and Business University Research Ethics Committee, reference number: No. (63) 2024).

### 2.3 Sample preparation

Sample extraction was performed using a previously described procedure.(Bian et al., 2022) Each CRP sample was ground using a grinder (S1-M83, Joyoung) and sieved through 40-mesh. Then, 100 mg of the CRP sample was precisely weighed and extracted using 500 μL of a solvent mixture consisting of a MeOH:ACN:water solution at a ratio of 2:2:1. The solution was vortexed for 1 min, ultrasonicated (JXD-02, Beijing Jinxing Ultrasonic Equipment Technology Co., Ltd.) for 30 min at room temperature, and stored at −20 °C for 1 h. Then, the mixture was centrifuged (14,000 *g*, 4 °C, 20 min), after which the supernatant was vacuum-dried. Next, 100 μL of an ACN aqueous solution (ACN:water = 1:1, v/v) was added, vortexed, and centrifuged (14,000*g*, 4 °C, 15 min). The supernatant was passed using a 0.22 nylon filter membrane and kept at 4 °C for subsequent mass spectrometry analysis. Six replications were performed.

### 2.4 Non-targeted metabolomics analysis

The samples were analyzed via UHPLC (1290 Infinity LC, Agilent Technologies) coupled to a quadrupole time-of-flight (AB Sciex Triple TOF 6600). UHPLC with a C-18 column (Waters, ACQUITY UPLC BEH C-18, 1.7 μm, 2.1 mm × 100 mm column) was used for sample separation at a column temperature of 40 °C, a flow rate of 0.4 mL/min, and an injection volume of 2 μL.

Mobile phase A consisted of 25 mM NH_4_AC and 0.5 % FA in water, while mobile phase B comprised MeOH. The gradient elution procedure included 5 % B for 0–0.5 min and a linear B increase to 100 % from 0.5 min to 10 min. Then, 100 % B was maintained from 10 min to 12 min, after which it was linearly decreased to 5 % from 12 min to 12.1 min, where it was maintained from 12.1 min to 16 min. The samples were placed in an automatic sampler at 4 °C for analysis. A random sequence was used for sample analysis to compensate for the fluctuation of the instrument. The quality control (QC) samples are inserted into the sample queue to monitor and evaluate the stability and reliability of the data.

The samples were analyzed using UHPLC-Q-TOF in both positive and negative ion modes. The ESI source conditions after chromatographic separation included an ion source Gas1 of 60, an ion source Gas2 of 60, a curtain gas of 30, a source temperature of 600 °C, an ionspary voltage floating (ISVF) of ±5500 V (positive and negative modes), an MS precursor ion MS^2^ ion scanning range from 60 Da to 1000 Da, a product ion scan *m/z* range of 25 Da to 1000 Da, a TOF MS scan accumulation time of 0.20 s/spectra, and a product ion scan accumulation time of 0.05 s/spectra. Secondary mass spectrometry was performed using information-dependent acquisition (IDA) in high sensitivity mode with a declustering potential (DP) of ±60 V (positive and negative modes) and a collision energy of 35 ± 15 eV. The IDA settings excluded isotopes within 4 Da and monitored 10 candidate ions per cycle.

### 2.5 Molecular networking analysis

A molecular network was created using the FBMN workflow (Nothias et al., 2020) on the GNPS platform (https://gnps.ucsd.edu) (Aron et al., 2020). The raw data files were converted to the GNPS compatibility format using MS-DIAL 4.80 and submitted to the FBMN platform using WinSCP to generate an MS/MS molecular network. Both the workflow precursor and fragment ion mass tolerance were set at 0.02 Da, while a cosine score above 0.7 was used to create the molecular network. More details about the FBMN results are available at http://gnps.ucsd.edu/ProteoSAFe/status.jsp?task=fe1a8418c3cc4afe9049e8ef8e0dccc1in the negative mode and https://gnps.ucsd.edu/ProteoSAFe/status.jsp?task=3a136f8c905242afa3986171f3404ffa in the positive ion mode. At least six matched fragment ions were required. Cytoscape 3.9.1 was used for network visualization (Otasek et al., 2019).

### 2.6 Metabolomics data processing

The raw data files obtained via UHPLC-Q-TOF-MS were used for peak alignment, retention time correction, peak area extraction, *m/z* value identification, binding ion addition, and compound name determination using the MS-DIAL 4.80 software (Tsugawa et al., 2020). QC samples were prepared to evaluate the reliability and reproducibility of the analytical results. The samples were mixed and assayed in sextuplicate. Total ion chromatogram overlay analysis, Pearson correlation analysis, multivariate control analysis, and relative standard deviation analysis of the QC samples (Fig. S2), as well as total ion chromatogram overlay analysis and Hotelling's T2 test of the whole samples (Fig. S3), demonstrated the excellent reproducibility and reliability of the analytical data. The SIMCA-P version 13.0 software (Umetrics, Malmö, Sweden) was used for principal component analysis (PCA), partial least-squares discriminant analysis (PLS-DA), and VIP score determination. SPSS 23 (SPSS 23.0; IBM Corp, NY, USA) was performed for ANOVA and employed to screen the differential metabolites, the ChiPlot (https://www.chiplot.online/) was used for the related heat map visualization and volcano map, the VirtualTaste tool (https://insilico-cyp.charite.de/VirtualTaste/) was used to predict the taste trait of compounds. The metabolites that differ among all samples were assessed via MetaboAnalyst 5.0 (https://www.metaboanalyst.ca/) with *Arabidopsis thaliana* as the background for the functional annotation and enrichment analysis of those present during the CRP aging process. Significant differences were calculated using the SPSS 23 software.

## 3 Results and discussion

### 3.1 Analysis of the CRP taste quality at different aging stages

**Fig. 1:**
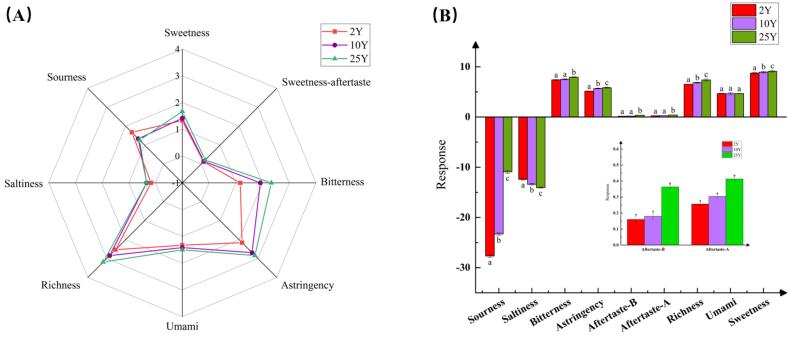
The sensory evaluation results of the CRP with different aging periods. (A) Radar plot. (B) Bar chart. Values of the same parameters with different letters (a-c) indicate significant differences (*p* < 0.05). (Aftertaste-A and aftertaste-B represent aftertaste- astringent and aftertaste-bitterness, respectively).

Our previous study analyzed the volatile aroma components in CRP at different aging stages, while the present study focused on its taste. Fig. 1A showed the QDA results of three aged CRP samples after assessment by a panel of 12 trained evaluators. An extended aging time enhanced the sweetness, bitterness, astringency, and richness of the aged CRP (*p* < 0.05) and decreased the sourness (*p* < 0.05), while no significant changes were evident in the sweetness-aftertaste, umami, and saltiness. The electronic tongue is based on a multi-channel sensor array working in concert to convert chemical signals into electrical signals to provide more objective flavor information. Fig. 1B presented the *E*-tongue results. Since the sourness and saltiness of the different aged CRP samples were below the human detection thresholds, these values were excluded from the range of valid taste indicators. The bitterness, astringency, after-bitterness, after-astringency, richness and sweetness increased as the aging time was extended (*p* < 0.05), while the differences in the development of the umami in the samples were insignificant, which was consistent with the sensory evaluation results. As the aging time was extended, the characteristic taste components, such as phenols and flavonoids, gradually accumulated in the CRP while the bitterness and astringency increased. Furthermore, microorganism activity and enzymatic reactions possibly caused sugar substance transformation and accumulation in the CRP while increasing the perception of the peel sweetness (Sun et al., 2023). As the aging period increased, the CRP exhibited more pronounced richness in the coordinated presentation of the various flavor components, resulting in a more intense, denser overall flavor.

### 3.2 Overall metabolomic analysis of the CRP at different aging stages

Non-targeted UHPLC-Q-TOF-MS was combined with a metabolomics method to profile the relationship between the metabolite fingerprint profiles and flavor. Most of the compound ions in the different aged CRP samples were separated within 16 min in the optimized UHPLC-Q-TOF conditions. This study annotated 1092 compounds by combining the FBMN platform and self-built databases (Fig. 4A), of which 626 metabolites were identified in positive ion mode and 466 in negative ion mode. These metabolites included 360 lipids and lipid-like molecules, 193 flavonoids, 148 organic acids and derivatives, 15 amines, 62 carbohydrates and carbohydrate conjugates, 20 coumarins and derivatives, 22 phenols, 24 alkaloids and derivatives, 46 benzene and substituted derivatives, 14 lignans, neolignans, and related compounds, 24 benzenoids, 9 nucleosides, nucleotides, and analogs, 23 organic oxygen compounds, 5 organic nitrogen compounds, 96 organoheterocyclic compounds, and 31 phenylpropanoids and polyketides.

FBMN is an advanced processing method for distinguishing isomers with similar secondary mass spectra. FBMN was used to cluster the secondary mass spectra of the CRP samples into molecular families according to similarity in the MS^2^ fragmentation pattern. These compounds were clustered and annotated by matching them with MS/MS fragment ions in the FBMN database, allowing for the identification of compounds of the same type (Li, Wu, et al., 2024). The cluster advantage of FBMN was employed to annotate unknown compounds by comparing the secondary mass spectra of annotated compounds with the corresponding compounds in the FMBN database. This approach compensated for the insufficient compound identification to database constraints. In negative ion mode, the molecular family contained 25,503 nodes ([M+H-H_2_O]^+^, [M+Na]^+^, [M-H+2Na]^+^, [M+H]^+^, and [M+NH_4_]^+^), consisting of 293 clusters (nodes ≥2) (Fig. S4). In positive ion mode, the molecular family included 21,414 nodes ([M-H]^−^, [M+HCOO]^−^, [M+FA-H]^−^, [M-CH_3_]^−^), consisting of 487 clusters (nodes ≥2) (Fig. S4). Flavonoids represented the dominant family of molecules in the molecular network of the CRP aged for different periods. The secondary mass spectrometry information of the flavonoids was manually annotated to verify the annotation accuracy of these constituents.

### 3.3 Molecular families and metabolite annotation

#### 3.3.1 Annotation of the flavonoids

**Fig. 2:**
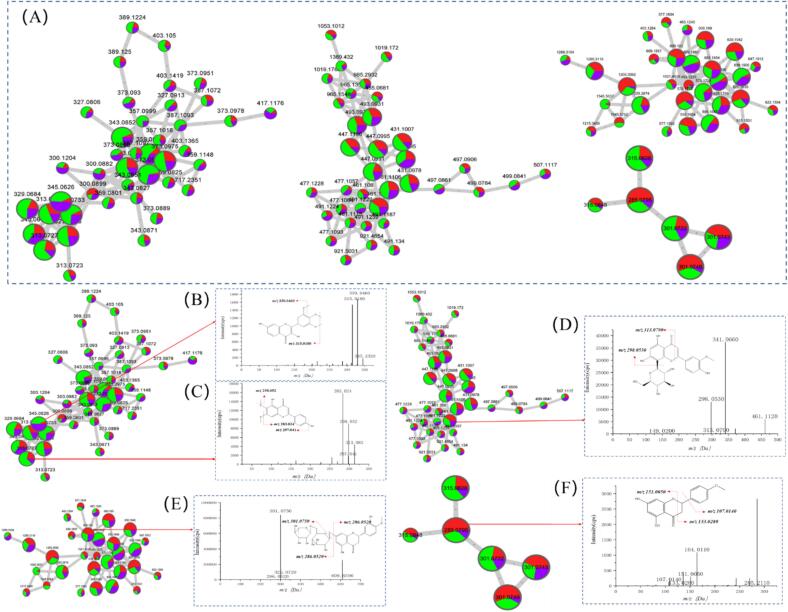
The molecular flavonoid families and structure deduction via MS^2^ spectrometry. (A) Molecular flavonoid family. (B) 5,7,3′,4′-Tetrahydroxy-6,8-dimethoxyflavone. (C) Cirsimaritin. (D) Scoparin. (E) Hesperidi. (F) Isosakuranetin. Red denotes 2Y, purple signifies 10Y, and green represents 25Y. Different color ratios in the nodes of the molecular family indicate the relative CRP abundance at different aging stages. (For interpretation of the references to color in this figure legend, the reader is referred to the web version of this article.)

The flavonoids primarily exhibited mild astringent and bitterness properties, which contributed significantly to the overall flavor of CRP. Flavonoids influence food flavor via interactions with other components, while their bioactive functions present various health benefits. Fig. 2 showed the molecular families of the five flavonoids and the inferred structures of some identified flavonoids under MS^2^ spectroscopy. Fig. 2A presented the different molecular flavonoid families, showing the identification of 43 flavonoids (larger nodes). The flavonoid MS^2^ data were manually analyzed to verify the annotation accuracy of these fractions. Fig. 2B–F presented the manually validated MS^2^ profiles of 5,7,3′,4′-tetrahydroxy-6,8-dimethoxyflavone, cirsimaritin, scoparin, hesperidin, and isosakuranetin, respectively.

Compound 1 (Fig. 2B) belonged to the same molecular family as compound 2 (Fig. 2C). The precursor ion *m/z* 345.2320 stripped of -CH_3_ (14 Da) yielded a characteristic fragment at *m/z* 330.0460, while the consecutive loss of methyl-neutrality formed *m/z* 315.0180, which was preliminarily identified as 5,7,3′,4′-tetrahydroxy-6,8-dimethoxyflavone. The parent ion of compound 2 (Fig. 2C) was *m/z* 313.0831, which displayed a similar bond-breaking pattern as compound 1. It's MS^2^ fragment underwent -CH_3_ stripping with -OCH_3_ to produce *m/z* 298.0523 [M-H-15]^−^ and *m/z* 283.0243 [M-H-30]^−^ MS^2^ ions. According to these characteristics, the compound was initially identified as cirsimaritin (Borrás Linares et al., 2011).

The parent ion of compound 3 (Fig. 2D) was *m/z* 461.1120, the MS^2^ fragment of which underwent a Retro-Diels-Alder (RDA) reaction to produce the *m/z* 313.0790 [M-H-148]^−^ fragmentation ion. The characteristic ion fragmentation of *m/z* 298.0530 could be attributed to the neutral loss of rhamnoside fragments (−146 Da). According to these characteristics, the compound was initially identified as scoparin (Koolen et al., 2013).

Compound 4 (Fig. 2E) was identified as hesperidin, which mainly displayed neutral glycoside loss fragments (−301 Da) with characteristic fragment ions at *m/z* 301.0750 and *m/z* 286.0520 (Nectoux et al., 2019). Compound 5 (Fig. 2F) displayed deprotonated *m/z* 285.2110 [M-H]^−^ ions in its mass spectrum. The MS^2^ cleavage pattern yielded two significant characteristic ions at *m/z* 151.0050 and *m/z* 133.0280, which were attributed to RDA fragmentation on the C-ring. From the elution order during reverse-phase chromatography, compound 5 was identified as isosakuranetin (Silberberg et al., 2006).

#### 3.3.2 Extrapolation of the unknown flavonoids

Previous studies have identified relatively small amounts of non-volatile metabolites in CRP, emphasizing the importance of appreciating its chemical composition more comprehensively, especially regarding unknown metabolites. This issue was addressed using FBMN, which utilized the MS^2^ fragmentation patterns to rapidly cluster molecular families and remove duplicate compounds with similar structures. As illustrated in Fig. 2, the known flavonoids were used as “seeds” to determine the potential existence and identification of unidentified components. This approach proved instrumental in enhancing the understanding of the chemical composition of CRP and provided compelling justification for further research and applications.

**Fig. 3:**
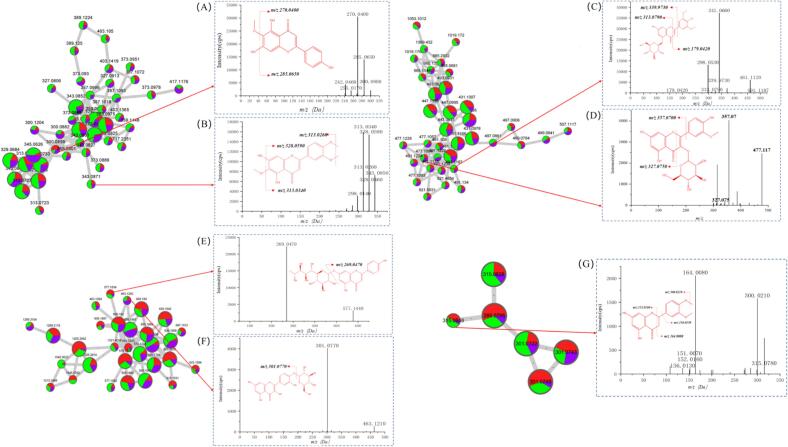
The inferred flavonoids absent from the FBMN database. (A) Hispidulin. (B) Eupatilin. (C) Tricin 5-glucoside. (D) Isorhamnetin 3-O-glucoside. (E) Rhoifolin. (F) Spiraeoside. (G) 5,7-dihydroxy-3′,4′-dimethoxyflavanone.

The GNPS spectral library successfully identified compound 1 as 5,7,3′,4′-tetrahydroxy-6,8-dimethoxyflavone (Fig. 2B) and compound 2 as cirsimaritin (Fig. 2C), while it showed that compound 6 (Fig. 3A) and compound 7 (Fig. 3B) shared a similar cleavage pattern. Compound 6 displayed *m/z* 300.0800 precursor ion, which yielded the characteristic *m/z* 270.0400 and *m/z* 285.0650 ion fragments. Therefore, it was tentatively inferred that compound 6 (*m/z* 300.0800) had lost both the methoxy group (–OCH_3_) and the hydroxyl group (–OH), leading to the conclusion that it was hispidulin (Fig. 3A). Similarly, compound 7 was preliminarily identified as eupatilin (Fig. 3B) (Wang et al., 2018).

The parent ion of compound 8 was *m/z* 491.1187, the MS^2^ fragments of which underwent an RDA reaction to produce the *m/z* 313.0790 [M-H-148]^−^ and *m/z* 179.0420 [M-H-312]^−^ fragment ions. The *m/z* 339.9730 ion fragmentation indicated the loss of the flavonoid B-ring, consequently identifying compound 8 as tricin 5-glucoside (Fig. 3C). Compound 9 displayed a similar cleavage pattern, yielding the characteristic *m/z* 357.0700 [M-H]^−^ and *m/z* 327.0750 [M-H-312]^−^ ion fragments, which were identified as isorhamnetin 3-O-glucoside (Fig. 3D).

The neutral loss fragmentation of the glycosides exhibited by compounds 10 (Fig. 3E) and 11 (Fig. 3F) yielded the characteristic *m/z* 269.0470 and *m/z* 301.0770 ion fragments, respectively, which were identified as rhoifolin and spiraeoside according to their respective retention times and comparison with the literature. Compound 12 (Fig. 3G) exhibited an RDA bond-breaking reaction, which generated characteristic fragments at *m/z* 152.0160 and *m/z* 164.0080. The loss of the B-ring produced a fragment ion at *m/z* 136.0130, which was tentatively identified as 5,7-dihydroxy-3′,4′-dimethoxyflavanone.

### 3.4 Analysis of the differential metabolites identified in the CRP aged for different stages

**Fig. 4:**
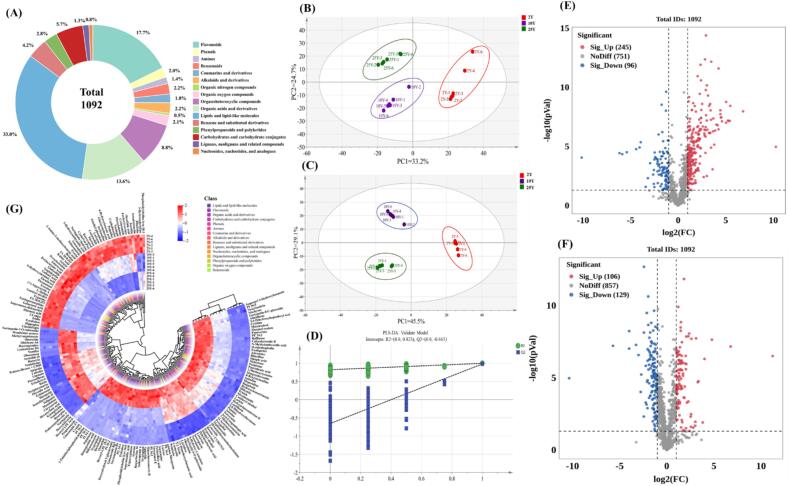
(A) Overall metabolite classification and percentage content. The multivariate data analysis of the CRP at different aging stages using UHPLC-Q-TOF-MS. (B) The PCA score plot; (C) The PLS-DA score plot; (D) The PLS-DA permutation test plot. The volcanic map of differential metabolites. (E) 2Y VS 10Y. (F) 10Y VS 25Y. (G) The Heat map of differential metabolites.

To comprehensively investigated the metabolic changes of CRP during aging, a multifactorial statistical analysis combining UHPLC-Q-TOF-MS untargeted metabolomics was employed to elucidate the dynamics of the key metabolites during the CRP aging process. The PCA score plot indicated cluster formation in all six replicates of each group, with significant separation between the clusters of the samples aged for different periods (Fig. 4B). The cumulative contribution of the first two principal components to the total variance was 57.9 % (PC1 = 33.2 %, PC2 = 24.7 %), indicating distinct differences between the samples and that the model effectively distinguished between the samples aged for different periods.

Furthermore, a supervised PLS-DA based chemometrics algorithm was applied to visually differentiate between the CRP samples in different aging stages, which was mainly used to separate the critical metabolites (Zhang et al., 2023). The results showed a distinct differentiation between the CRP aged for different stages, with R2Y and Q2Y ratings of 0.986 and 0.970, respectively. This suggested that the model displayed excellent interpretive and predictive abilities. The PLS-DA score plot demonstrated effective sample clustering into three discernible groups. The results indicated that component 1 accounted for 45.5 % of the observed variance, while component 2 explained 29.1 % (Fig. 4C). The PLS-DA score plot exhibited a distinct separation between the aging CRP samples. The reliability of the PLS-DA model was verified by conducting 200 test iterations (Fig. 4D). The exchange model was evaluated against the original un-exchanged model considering both self-prediction and cross-validation, which demonstrated the Q2 exchange test values were inferior to the original model intercepts and R2. Additionally, the Q2 regression line related to the vertical axis fell below zero, demonstrating that the model exhibited robust performance and was not overfitted. The significance of each metabolite in enhancing the discriminative power of the PLS-DA model was assessed by calculating the VIP value, combined with the volcano plot analysis (Fig. 4E–F), 189 potentially differential metabolic components (VIP > 1, *p* < 0.05 and FC > 1.50 or FC < 0.67) were ultimately identified in 15 categories that were screened to identify differences between CRP aging processes (Table S3). The differential metabolites of the CRP of different ages were further visualized using a heat map (Fig. 4G), which primarily including phenols and flavonoids, carbohydrates and carbohydrate conjugates, organic acids and derivatives and others.

### 3.5 Dynamic changes of taste compounds during the storage

**Fig. 5:**
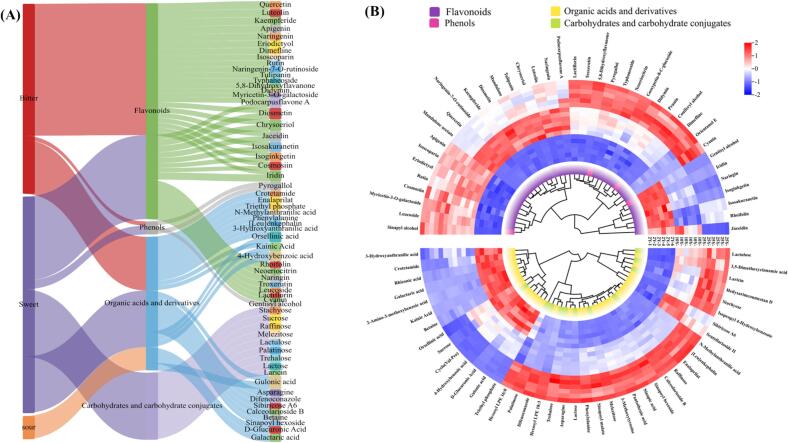
(A) The sankey diagram for predicting the taste potential of different metabolites based on the virtual taste tool. (B) The clustering heatmap for predicting taste attribute metabolites based on virtual taste tools. (Flavonoids and phenols (top), organic acids and derivatives and carbohydrates and carbohydrate conjugates (bottom)).

In this study, the VirtualTaste tool was used to evaluate the flavor properties of different components in four major classes of compounds in differential compounds, phenols, flavonoids, carbohydrates and carbohydrate conjugates, and organic acids and derivatives (Fig. 5A, Table S4).

#### 3.5.1 Phenols and flavonoids

CRP is a rich source of phenolic compounds, particularly flavonoids. Flavonoids exhibit lower thresholds and represent the primary non-volatile flavor components contributing to the bitterness and velvety astringency, which is crucial for determining overall quality (Wang, Qu, et al., 2024). The flavonoid metabolite content in CRP initially increases, followed by a decrease with extended aging time. In contrast, most phenolic acids with antioxidant and anti-inflammatory activities tend to increase during the aging process (Luo et al., 2019). This study identified 35 different flavonoid compounds. Using the VirtualTaste tool to investigate the taste prediction potential of differential phenols and flavonoids compounds, we discovered that 24 metabolites among these compounds are primarily associated with bitterness (Fig. 5A, Table S4). The levels of sinapyl alcohol, coniferyl alcohol increased notably with age (Fig. 5B). As the lignin precursors, the increased content of sinapyl alcohol and coniferyl alcohol drive the flavor shift from fruity to woody, and enhanced the mellow taste. Various flavonoid compounds leading to quality differences among CRP samples with different aging degrees, including naringenin, eriodictyol, quercetin, luteolin, and apigenin, their contents exhibit an initial increase followed by a decrease with the extension of the aging period (Fig. 5B). Naringenin exhibits a strong correlation with bitterness (p_bitter_ = 0.948). By interacting with sweet components in the taste profile of aged CRP, it achieved flavor balance and enriched the complexity of the palate. Eriodictyol (p_bitter_ = 0.891) and apigenin (p_bitter_ = 0.998), while imparting bitterness to CRP, simultaneously endow it with a herbal aroma. The floral aroma of luteolin effectively neutralizes the bitterness (p_bitter_ = 1.000) of CRP, making the overall taste become softer. Hydrolysis of flavonol glycosides elevates flavonol aglycone levels, particularly quercetin, which imparts bitterness (p_bitter_ = 1.000) and astringency to CRP, can interact with other components, such as organic acids, to further enhance the mellow flavor (Fu et al., 2025). The changes of these metabolites during the aging period of CRP made the taste of CRP gradually transform from floral and fruity aromas to woody herbal aroma, which was consistent with our previous findings (Jiang et al., 2023). Additionally, the content of flavanol glycosides, especially naringenin-7-glucoside (p_bitter_ = 0.746) and myricetin-3-O-galactoside (p_bitter_ = 0.537), was critically important for the astringency of tea and enhancing the bitterness of caffeine.

#### 3.5.2 Carbohydrates and carbohydrate conjugates

The differential carbohydrates and carbohydrate conjugates in the CRP primarily consisted of soluble sugars. The soluble sugars in tea infusions impart a sweet flavor and are also essential for the creation of volatile metabolites (Wang, Feng, et al., 2024). This study identified 13 different Carbohydrates and carbohydrate conjugates. The VirtualTaste based prediction revealed that 9 metabolites exhibited primary associations with sweetness (Fig. 5A, Table S4), among which stachyose, sucrose, raffinose, lactulose, and trehalose represented the predominant differential metabolites. Sucrose (P_sweet_ = 1.000) exhibited a sustained decline during aging, which was probably resulted from a combination of chemical and biological processes such as glycolysis. And the stachyose (p_sweet_ = 1.000), raffinose (p_sweet_ = 1.000), lactulose (p_sweet_ = 0.996), and trehalose (p_sweet_ = 0.992) exhibited a significant increase during aging (Fig. 5B). Carbohydrates degrade or transform during the aging process, generating new sweet substances. These substances interact with the original soluble sugars to gradually enhance the sweetness of the peels during the aging process. Their synergistic interaction with other flavor components balances the CRP flavor (Shan et al., 2024). In addition, these carbohydrates and carbohydrate conjugates provide the substrate foundation for flavonoid glycoside formation.

#### 3.5.3 Organic acids and derivatives

Organic acids are vital intermediate metabolites in the tricarboxylic acid cycle and the manganic acid pathway and are crucial for tea flavor. Organic acids contribute to the acidic and fruity taste of tea while reducing bitterness (Wang, Feng, et al., 2024). Among 30 differential organic acids and derivatives metabolites in CRP across aging periods, 19 taste-active compounds were discerned through VirtualTaste analysis (Fig. 5A, Table S4). The data indicated a decline in the concentrations of organic acids, such as gulonic acid (p_sour_ = 0.968), d-glucuronic acid (p_sour_ = 1.000), and galactaric acid (p_sour_ = 1.000) as aging progressed (Fig. 5B). The d-glucuronic acid naturally existed in citrus pectin polysaccharides and was released during pectin degradation, directly contributing a weak sour taste. It synergistically enhanced astringency with flavonoids, while part of it was metabolized by microorganisms, reducing acidity and gradually highlighting sweetness. The galactaric acid mainly originated from galactose oxidation or pectin degradation. As aging time prolonged, it slowly transformed into low molecular weight acids, further softening the sourness and synergistically enhancing the aftertaste with alkaloids. The gulonic acid content decreased with prolonged aging time, nearly eliminating sourness and astringency (Tan et al., 2024). During the aging process, these organic acids experienced intricate biochemical transformations that contributed to a reduction in the acidity of the CRP, which reduced the peel acidity, while interacting with other compounds to increase the flavor complexity and balance in the long-aged CRP.

### 3.6 Analysis of the metabolic pathways

Metabolomics can offer valuable insight into the biosynthetic and degradative pathways, as well as the translational and regulatory relationships between metabolites in the CRP aging process due to various stressors. To thoroughly examine the various metabolic pathways during the CRP aging process, this study conducted the KEGG enrichment analysis of the 189 identified differential metabolites. Furthermore, the functional KEGG pathways were explored to reveal those relevant to this process. MetaboAnalyst offers a powerful platform for examining metabolic pathways and currently supports 25 metabolomics data modalities. Given the scarcity of information regarding the CRP species during the metabolic pathway analysis using the MetaboAnalyst platform, *Arabidopsis thaliana* was selected as the most suitable alternative environment.

**Fig. 6:**
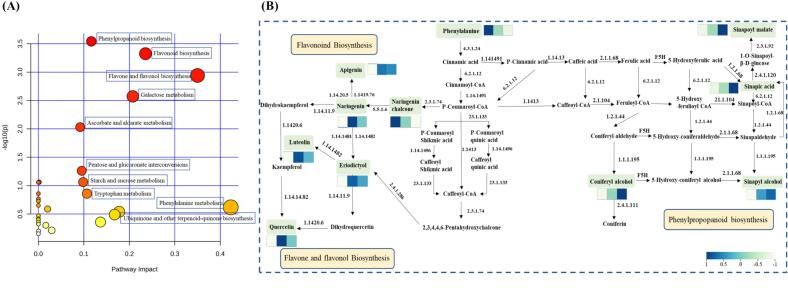
(A) The KEGG pathway analysis diagram of the key metabolic pathways. (B) The metabolic pathway schematic of Phenylpropanoid biosynthesis, Flavonoid biosynthesis, Flavone and flavonol biosynthesis.

An analysis of pathway enrichment was conducted utilizing the MetaboAnalyst tool to assess the alterations in metabolites throughout the aging process of CRP. The bubble chart illustrated in Fig. 6A depicted the metabolic pathways that were engaged in the CRP aging process. Each bubble within the chart stands for a specific metabolic pathway, with the horizontal axis values and the sizes of the bubbles reflecting the extent of influence observed in the topological analysis. Larger bubble sizes correspond to higher horizontal axis values, signifying a greater level of enrichment. The vertical axis values and the colors of the bubbles represented the *p*-values obtained from the enrichment analysis, where a darker hue indicated a lower p-value and greater significance of enrichment (Li, Wu, et al., 2023). The metabolic pathway analysis of the 189 differentiated compounds identified during the CRP aging process revealed that they were mainly involved in 10 key differentiated metabolic pathways, including phenylpropanoid biosynthesis, flavonoid biosynthesis, flavone and flavonol biosynthesis, galactose metabolism, ascorbate and aldarate metabolism, pentose and glucuronate interconversions, starch and sucrose metabolism, and tryptophan metabolism, phenylalanine metabolism, ubiquinone and other terpenoid-quinone biosynthesis.

The KEGG pathway database was searched manually to further explore the three most important differential metabolic pathways during the CRP aging process. Fig. 6B showed the systematic pathways of phenylpropanoid biosynthesis, flavonoid biosynthesis, and flavone and flavonol biosynthesis. P-Coumaroyl-CoA represented the key node connecting phenylpropanoid biosynthesis and flavonoid metabolism, and the core precursor of many flavonoids. The flavonoids were synthesized via the phenylpropanoid branch of metabolism. The phenylpropanoid formed chalcone in the presence of various enzymes, which was then isomerized to flavanone aglycones such as naringenin, hesperetin, and apigenin (Li et al., 2015). In addition, naringenin represented the key connection between the metabolic pathways of flavonoid biosynthesis and flavone and flavonol biosynthesis. The naringenin biosynthesis involved the conversion of via L-phenylalanine and L-tyrosine into p-coumaric acid via the shikimic acid pathway. Subsequently, sequential condensation with various enzymes resulted in naringenin chalcone formation (Liu et al., 2022).

L-phenylalanine, coniferyl alcohol, coniferin, sinapyl alcohol, sinapate, and sinapoyl malate represent the metabolic and key nodes in the phenylpropanoid biosynthesis metabolic pathway. Various phenolic compounds were involved in phenylpropanoid biosynthesis metabolism, including cinnamic acid, p-cinnamic acid, caffeic acid, and sinapic acid. Phenolic compounds constituted the predominant class of secondary metabolites in CRP, demonstrating potent antioxidant capacity and contributing to its characteristic flavor profile (Bian et al., 2022). Phenolic compounds primarily provide bitterness and astringency to food products. When polyphenols reacted with salivary proline, they precipitate on the surface of the mouth, leading to roughness on the tongue (Luck et al., 1994). Phenolics, such as lignin, represented the final metabolites of the phenylpropane synthesis pathway. Their distinctive presence in the stromal cells of the aged CRP was the primary factor responsible for their flavor. The levels of several key lignin intermediates, including sinapic acid and coniferyl alcohol, decreased gradually during the different aging periods. This demonstrated that the variation in the phenolic compounds among the peels at various aging stages contributed to the flavor differences (Zhang et al., 2024).

Flavonoids constituted a significant class of secondary metabolites biosynthesized via the phenylpropanoid pathway (Wang et al., 2021). This study found that the significant differences in flavonoids were mainly composed of flavonoid glycosides. These differential isoflavonoid compounds contributed significantly to the flavor variation among the CRP at different aging stages. The apigenin, naringenin, naringenin chalcone, eriodictyol, luteolin, and quercetin represented the metabolic and key nodes during flavonoid biosynthesis, while the apigenin, luteolin, and quercetins dominated during flavone and flavonol biosynthesis. Flavonoids and their glycosides were abundant polyphenolic sources, while the small-molecule compounds with low taste thresholds in the CRP mainly consisted of flavonoid ligands such as quercetin, apigenin, and naringenin, significantly contributing to the CRP flavor. The CRP aging process involved complex degradation, oxidation, and polymerization reactions due to physical, chemical, and biological factors (Yue et al., 2023). The cells in the CRP displayed no significant damage during the pre-aging period, while the physiological changes mostly involved enzymatic hydrolysis. The cellular protoplasmic membrane permeability increased during the subsequent aging stages, while oxidation was gradually enhanced. The aging process affected the chemical composition of the CRP, leading to the production of flavonoids either in their unbound state (glycosidic elements) or attached to sugar molecules. Flavonoid glycosides, primarily in the form of O-glycosides, represented the most prevalent glycosylated flavonoids. Glycosylation enhanced solubility, distribution, and metabolism by facilitating membrane transport. As the aging period was extended, the CRP chemical composition gradually stabilized, leading to flavor component accumulation (Dias et al., 2021). Food flavor represented a complex perceptual system. In CRP, metabolites contributed to flavor profiles through both gustatory mechanisms and synergistic interactions among chemical constituents. In conclusion, taste substance synthesis and degradation were primarily observed phenylpropanoid biosynthesis, flavonoid biosynthesis, flavone, and flavonol biosynthesis. This highlighted their pivotal role in mediating flavor compound changes.

## 4 Conclusion

In summary, this study comprehensively elucidated the distinctive metabolic characteristics of CRP during aging by employing untargeted metabolomics coupled with FBMN. It successfully identified key differential metabolites and further delineated the metabolic pathways underlying these differential compounds. Specifically, extending the aging time increased the bitterness, astringency, aftertaste-bitterness, aftertaste-astringency, and sweetness of the CRP while decreasing the acidity, which was consistent with the sensory evaluation results. Furthermore, 1092 metabolites were identified during the CRP aging process by combining non-targeted metabolomics and FBMN analysis. Of these, 189 differential metabolites are found to be involved in flavor formation during the CRP aging process via 10 primary metabolic pathways. It was also determined that the core pathway for flavor transformation is phenylpropane/flavonoid biosynthesis. The catalytic effect of endogenous enzymes and the active involvement and metabolism of microorganisms during the metabolic transformation process of CRPs lead to the production, transformation, and accumulation of metabolites, which increased the overall flavor. This study offered novel insights into the dynamics of metabolite profiles and sensory qualities during the CRP aging, and also established a data foundation for a deeper understanding of the unique flavor formation mechanism of CRP.

Additionally, this study identified various lipid metabolites based on non-target metabolomics. The substrates of lipid metabolism involved in the flavor formation of CRP, which are precursors of aromatic compounds, are under further investigation. Furthermore, given the complexity interactions among metabolites, microorganisms, and enzymatic activities during CRP aging, future research should focus on investigating the specific roles of these emerging components in flavor perception and nutritional contribution, elucidating molecular mechanisms underlying their interactions, and exploring genetic factors regulating the biosynthesis and functional activities of metabolites, microorganisms, and enzymes.

## Statement of informed consent

All participants in this study read the study description carefully before performing the sensory evaluation, agreed to the approval of the ethics committee and the use of sensory data, and signed an informed consent form.

## CRediT authorship contribution statement

**Kunli Xu:** Writing – review & editing, Writing – original draft, Software, Methodology, Investigation, Data curation, Conceptualization. **Sen Mei:** Writing – review & editing, Data curation, Conceptualization. **Jiahuan Liu:** Methodology, Data curation. **Zirui Guo:** Writing – review & editing, Conceptualization. **Fanyu Meng:** Writing – review & editing, Methodology. **Yanbo Wang:** Supervision. **Bei Wang:** Writing – review & editing, Supervision, Funding acquisition, Conceptualization.

## Ethics statement

This study recruited 12 trained panelists (4 males, 8 females, 28–42 years) to perform the sensory evaluation of CRP, and the Research Ethics Committee of Beijing Technology and Business University approved the research protocol. Reference number No. (63) 2024.

## Funding

Supported by the fund of Cultivation Project of Double First-Class Disciplines of Food Science and Engineering, Beijing Technology & Business University (BTBUYXTD202205).

## Declaration of competing interest

The authors declare that they have no known competing financial interests or personal relationships that could have appeared to influence the work reported in this paper.

## Data Availability

Data will be made available on request.
