# Supplementary material for: Revealing the dynamic changes in the metabolites and sensory quality of Citri Reticulatae Pericarpium during aging using feature-based molecular networks and metabolomics
Source: Food Chem X. 2025 Aug 7;29:102884. doi: 10.1016/j.fochx.2025.102884 (PMC12357287; doi:10.1016/j.fochx.2025.102884)
Supplement: Supplementary material 1 [file mmc1.docx]

**Revealing dynamic changes in metabolites and sensory quality of Citri Reticulatae Pericarpium during aging using feature-based molecular networks and metabolomics**

Kunli Xu^1,2^, Sen Mei^3^, Jiahua Liu^2^, Zirui Guo^2^, Fanyu Meng^1,2^, Yanbo Wang^1,2^, Bei Wang^1,2*^

1. Key Laboratory of Geriatric Nutrition and Health (Beijing Technology and Business University), Ministry of Education, Beijing 100048, China.

2. School of Food and Health, Beijing Technology and Business University, Beijing 100048, China.

3. State Key Laboratory of Food Science and Resources, Nanchang University, Nanchang, 330047, PR, China

Corresponding authors:
Bei Wang: Tel, 010-68984003; E-mail, wangbei@th.btbu.edu.cn

Fig. S1 PCA analysis of the E-tongue during the aging process of CRPs.

Fig. S2 Total ion chromatogram overlay analysis plot (A-B), Pearson correlation analysis plot (C-D), multivariate control analysis plot (E-F), relative standard deviation analysis plot (G-H) for QC samples. (A, C, E, G represent negative ion mode; B, D, F, H represent positive ion mode).

Fig. S3 Multiple peak detection overlay analysis plot (A-B), and Hotelling's T2 test plot (C-D) for the whole sample. (A, C represent negative ion mode; B, D represent positive ion mode).

Fig.S4. Molecular network of aged CRPs. In negative ion mode (A). In positive ion mode (B).


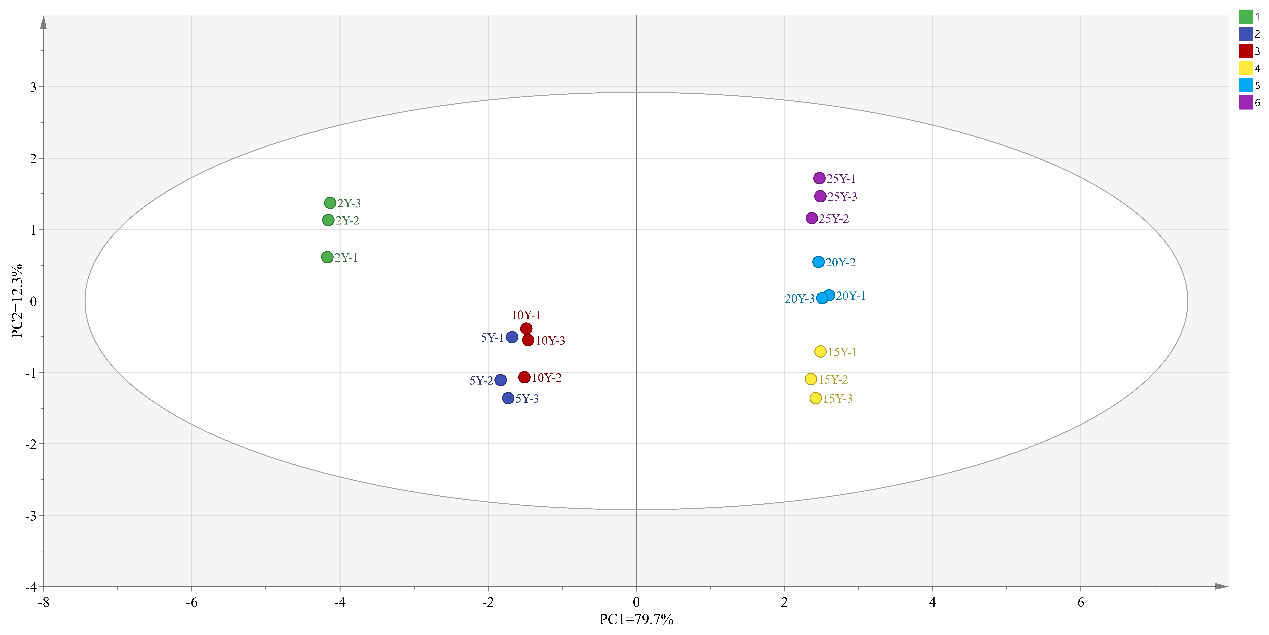


Fig. S1 PCA analysis of the E-tongue during the aging process of CRPs.

(A)
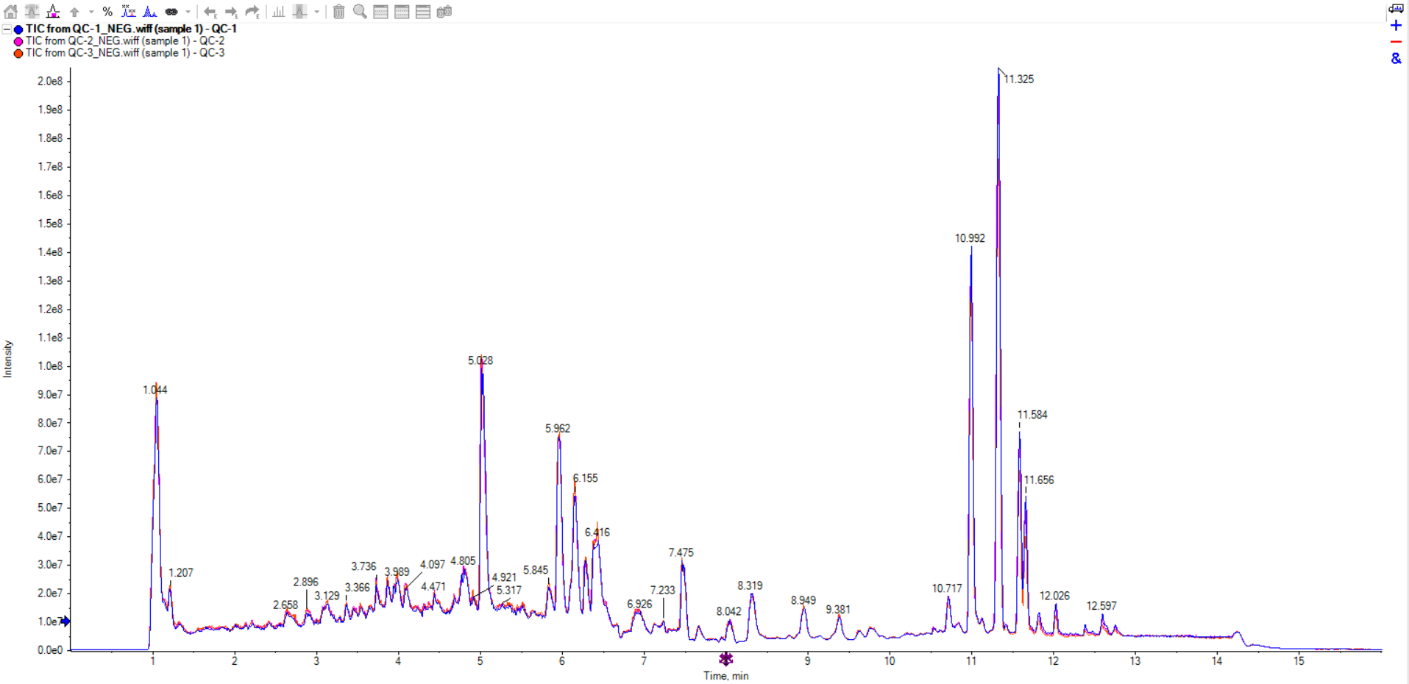


(B)


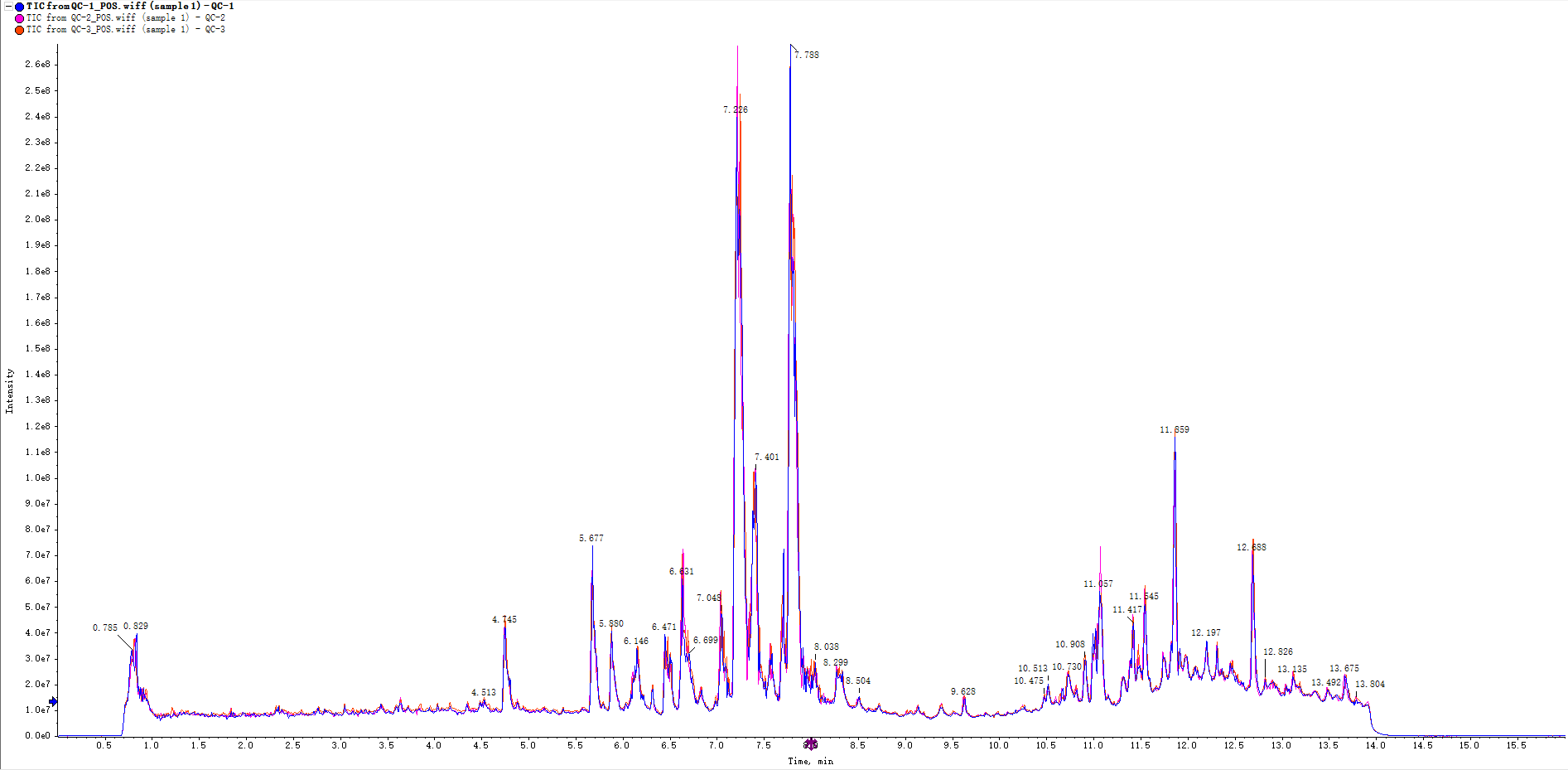


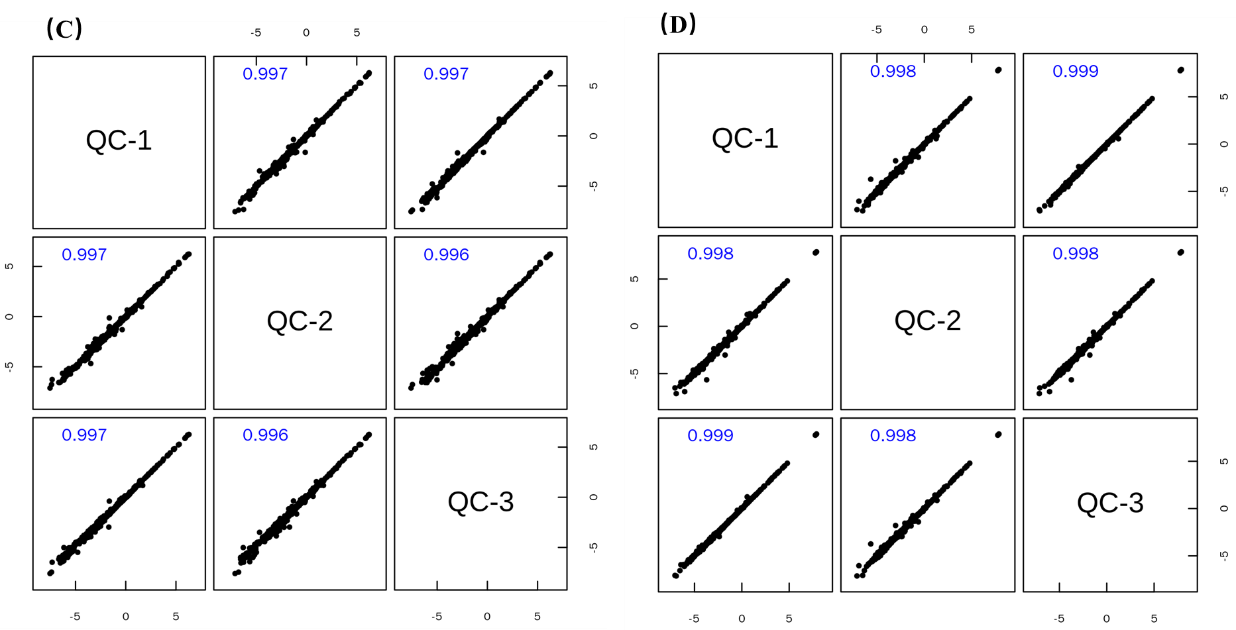


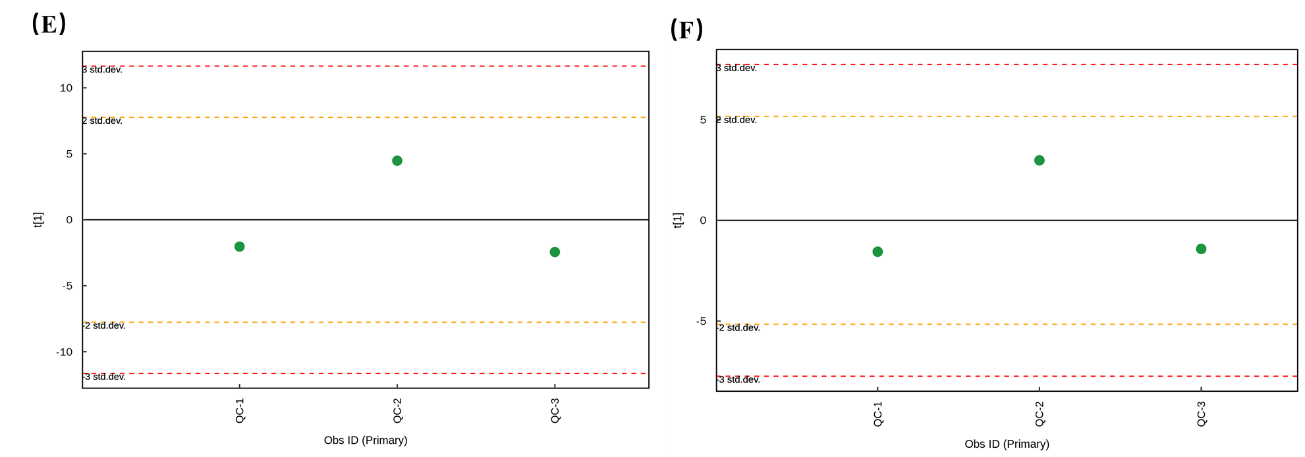


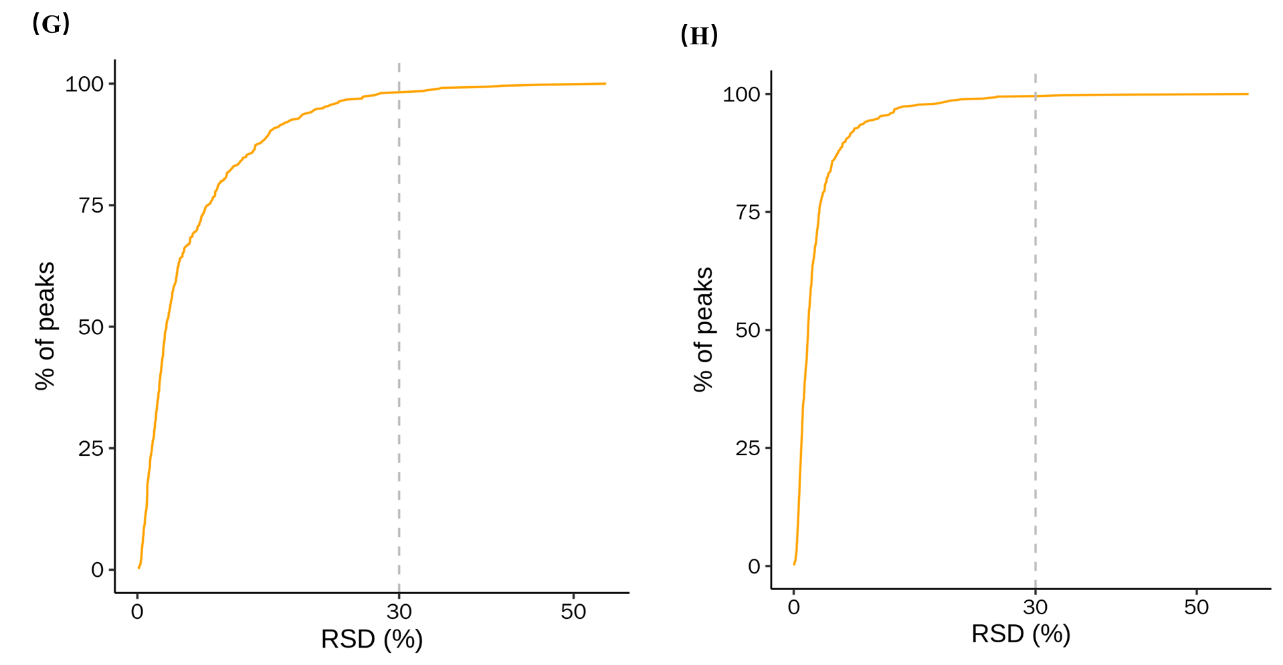


**Fig. S2** Total ion chromatogram overlay analysis plot (A-B), pearson correlation analysis plot (C-D), multivariate control analysis plot (E-F), relative standard deviation analysis plot (G-H) for QC samples. (A, C, E, G represent negative ion mode; B, D, F, H represent positive ion mode).

(A)


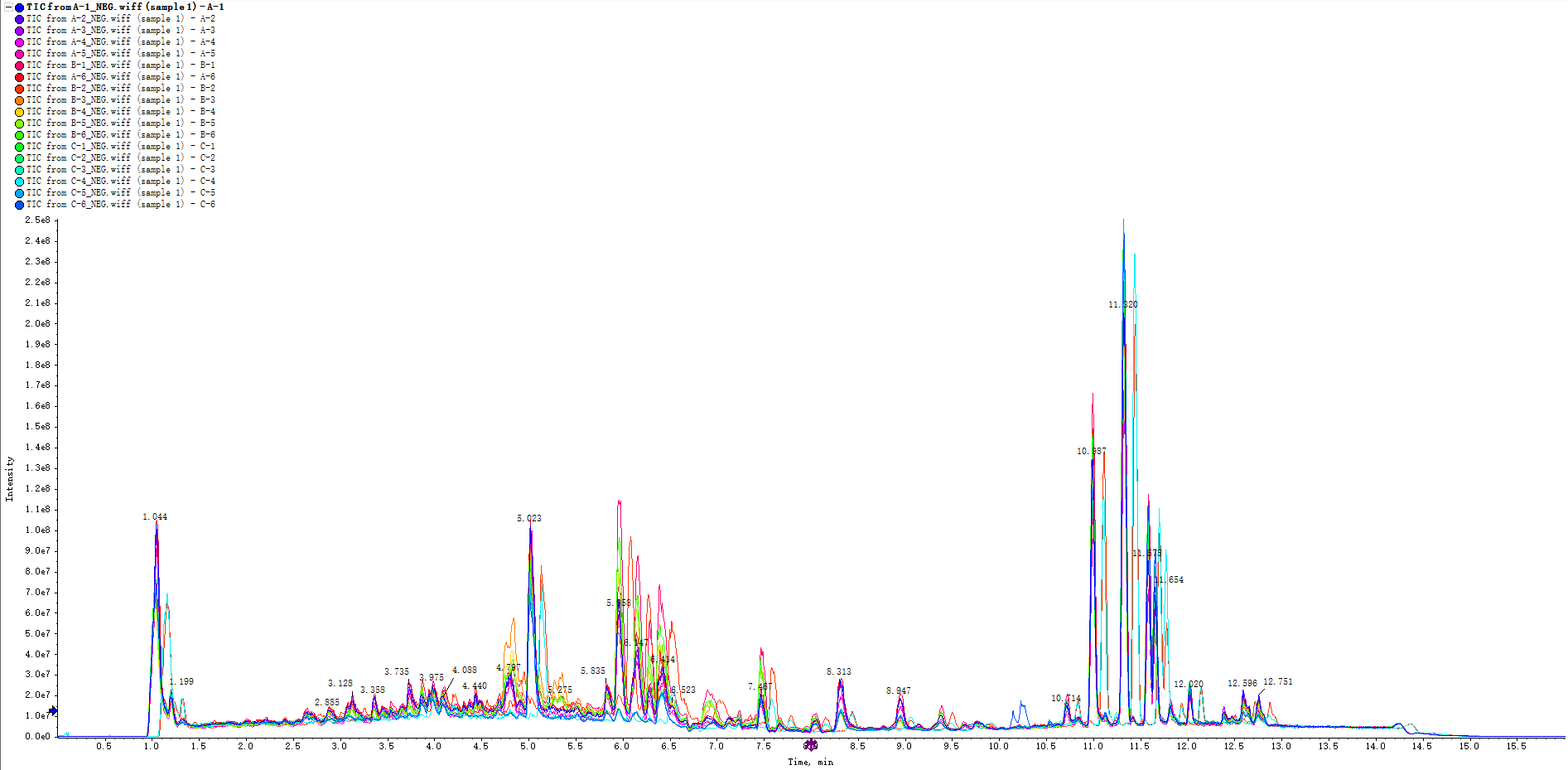


(B)


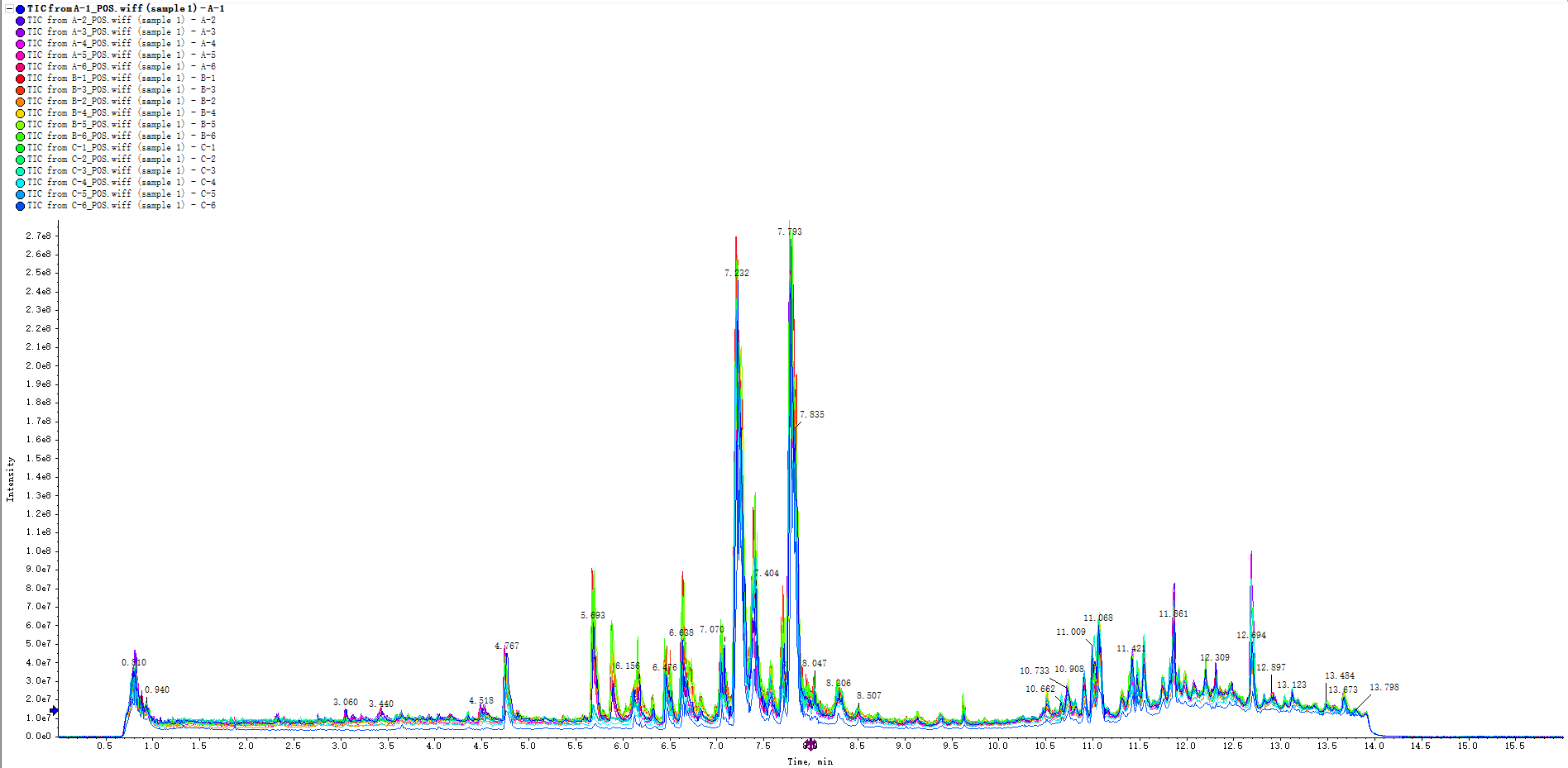


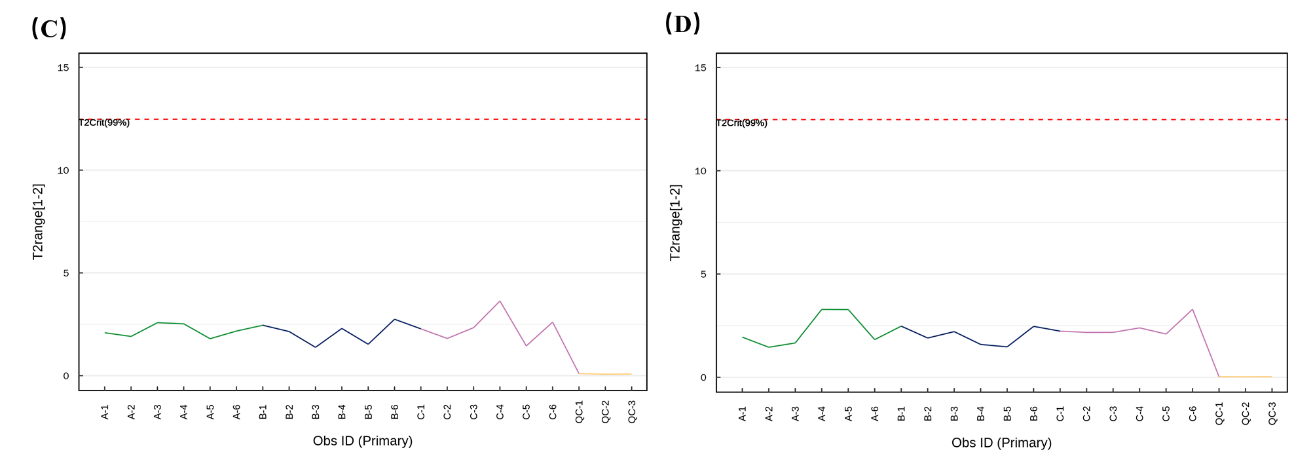


Fig. S3 Multiple peak detection overlay analysis plot (A-B), and Hotelling's T2 test plot (C-D) for the whole sample. (A, C represent negative ion mode; B, D represent positive ion mode).

(A)


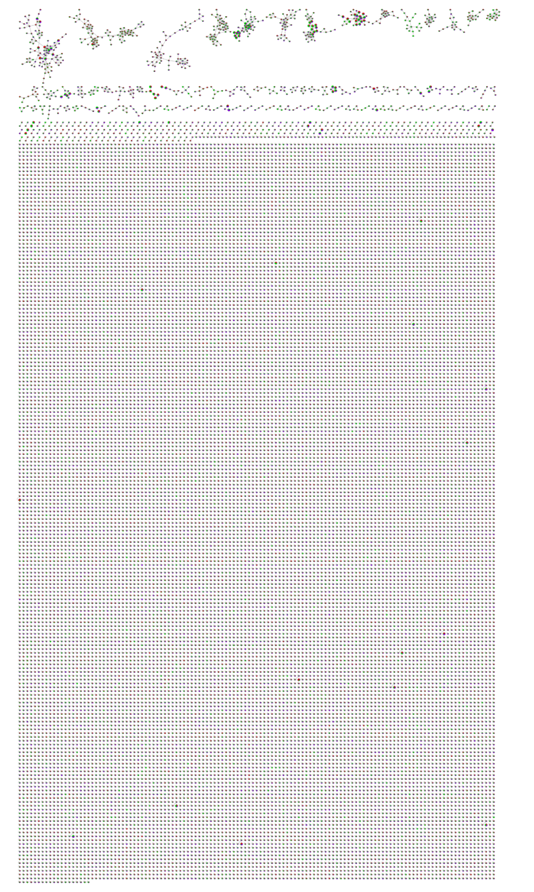


(B)


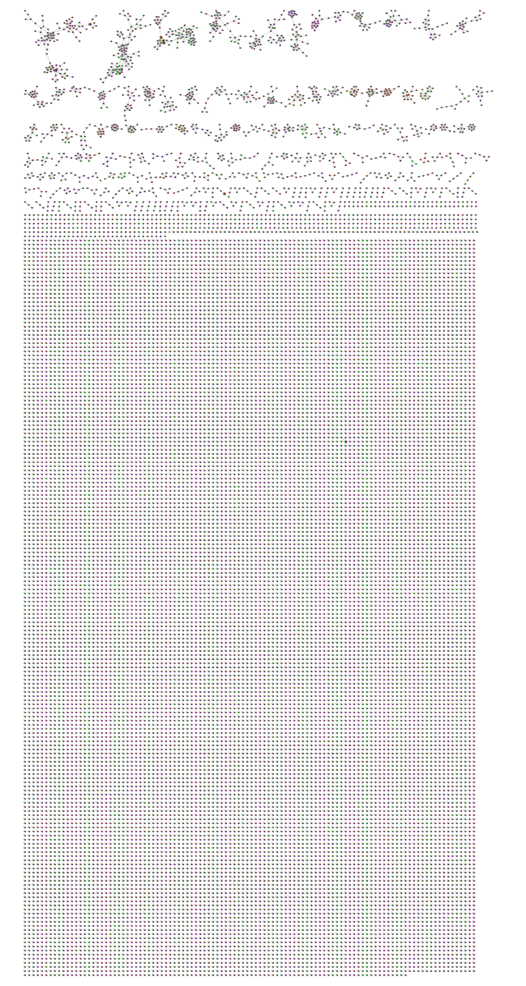


Fig.S4. Molecular network of aged CRPs. In negative ion mode (A). In positive ion mode (B).
